# Chr23-miR-200s and Dmrt1 Control Sexually Dimorphic Trade-Off Between Reproduction and Growth in Zebrafish

**DOI:** 10.3390/ijms26041785

**Published:** 2025-02-19

**Authors:** Si Ge, Ying Liu, Haoran Huang, Jiawang Yu, Xiaohui Li, Qiaohong Lin, Peipei Huang, Jie Mei

**Affiliations:** 1 Hubei Hongshan Laboratory, College of Fisheries, Huazhong Agricultural University, Wuhan 430070, China; si_ge3426@webmail.hzau.edu.cn (S.G.); liuying199705@126.com (Y.L.); yjw999@webmail.hzau.edu.cn (J.Y.); 2 School of Animal Science and Nutritional Engineering, Wuhan Polytechnic University, Wuhan 430023, China; 18171602049@163.com; 3 Yangtze River Fisheries Research Institute, Chinese Academy of Fisheries, Wuhan 430223, China; lixiaohui@yfi.ac.cn; 4 State Key Laboratory of Breeding Biotechnology and Sustainable Aquaculture, The Innovative Academy of Seed Design, Institute of Hydrobiology, Chinese Academy of Sciences, Wuhan 430072, China; qiaohonglin@ihb.ac.cn

**Keywords:** growth, reproduction, sexual dimorphism, trade-off, Dmrt1, miR-200

## Abstract

In animals, a trade-off exists between reproduction and growth, which are the most fundamental traits. Males and females exhibit profound differences in reproduction and growth in fish species. However, the precise molecular mechanism governing this phenomenon is still not clear. Here, we uncovered that chr23-miR-200s and *dmrt1* knockout specifically caused an impairment in reproduction and an increase in body growth in female and male zebrafish, respectively. Chr23-miR-200s and Dmrt1 directly regulate the *stat5b* gene by targeting its 3′UTR and promoter. The loss of *stat5b* completely abolished the elevated growth performance in chr23-miR-200s-KO or *dmrt1*^−/−^ zebrafish. Moreover, the *dmrt1* transgenic zebrafish had significantly lower body length and body weight than the control males, accompanied by a significant reduction in *stat5b* expression in the liver of transgenic fish. In summary, our study proposes a regulatory model elucidating the roles of chr23-miR-200s and Dmrt1 in controlling the sexually dimorphic trade-off between reproduction and growth.

## 1. Introduction

In most cases, genetic analysis of important traits mainly focuses on a single phenotypic trait. Recently, the trade-off relationships among different traits have been discovered [1,2]. The phenotype and genetic mechanism of trade-offs between disease resistance and high yield [3], spikelet number and fertility [4], growth and defense [5,6], growth and stress response [7], and reproduction and seed size–number [8] have been widely studied in plants. In addition, trade-offs have also been frequently observed in lower eukaryotes, including the trade-offs between sex and growth in diatoms [9], antibiotic tolerance and growth rate in bacteria [10], detoxification and reproduction, male success in sperm competition and offspring quality, gut homeostasis and lifespan, immunity and reproduction, and growth and immunity in insects [11,12,13,14,15,16,17]. The evolutionary trade-offs between offspring size and number across amphibians [18], as well as reproduction and life span in dogs [19], have been observed. Disruption in the trade-off between traits usually causes developmental disorders or diseases [20,21]. However, we currently have a very limited understanding on the regulatory mechanism of trade-offs between different traits in vertebrates.

Trade-offs in fish species have been found between physiological plasticity and growth [22], fish abundance and body size [23], muscular endurance and muscle mass or contractility [24], and pigmentation loss and adaptation to food-deprived caves [25]. Growth and reproduction are the most fundamental characteristics of organisms, which are intertwined and require a lot of energy [26]. The trade-off between growth and reproduction has been observed in fish; for example, GH-transgenic fish displayed a delay in gonadal development and impaired reproduction [27]. The trade-off between growth and reproduction is an essential part of the adaptive strategies for animal evolution [28]. Many studies have extensively explored the important genes and molecular pathways regulating reproduction or growth, but the molecular mechanism for trade-off between reproduction and growth is still unclear.

Sexual dimorphism refers to sex differences in many biological and physiological characteristics between males and females of the same species, such as sexual dimorphism in shape, growth, metabolism, reproduction, and disease resistance [29,30]. Sexual selection has been considered the main evolutionary force of sexual dimorphism and to be responsible for the macroevolution of gene expression [31,32,33], while sexual dimorphism is the consequence of sex-biased gene expression at different developmental stages [29,34]. Recent studies suggest that sexual differences in genetic and genomic architectures may contribute to sexual dimorphism in many complex traits [35,36,37,38]. However, the molecular mechanism of how sex-specific traits develop and evolve remains unclear.

The miR-200 family, a highly conserved group of microRNAs, is extensively expressed within the hypothalamic–pituitary–gonadal (HPG) axis in vertebrates [39,40]. Specifically, miR-200b and miR-429 play critical roles in mouse ovulation and are essential for female fertility [41]. In Japanese flounder, the miR-200 family targets *amh*, influencing gonadal development [42]. Similarly, the miR-200 cluster on chromosome 23 (chr23-miR-200s) regulates oocyte maturation and ovulation in female zebrafish [43]. Double-sex and Mab-3-related transcription factor 1 (*Dmrt1*) is a highly conserved gene involved in male sex determination and differentiation across various animal species [44]. In mice, *Dmrt1* is essential for maintaining testis identity, and loss of function allows sertoli cells to reprogram into granulosa cells. In zebrafish, *dmrt1* is required for the maintenance, self-renewal, and differentiation of male germ cells [45]. Despite these insights into reproduction, the regulatory mechanisms of miR-200 and *dmrt1* in growth-related processes remain poorly understood, with limited evidence available to elucidate their roles in these pathways.

In this study, we utilized zebrafish models with reproductive defects, specifically the chr23-miR-200s and *dmrt1* mutant lines, to investigate the relationship between reproduction and growth. Our primary objective was to elucidate the molecular mechanisms underlying the trade-off regulation between these two fundamental biological processes. Through comprehensive phenotypic analysis of growth-related traits in both mutant lines, we further identified and validated downstream target genes involved in growth regulation. Altogether, we found that the trade-off between growth and reproduction in female and male zebrafish was controlled by chr23-miR-200s and Dmrt1, respectively, opening new avenues for mechanistic research on sexually dimorphic regulation of the trade-off between growth and reproduction in animals.

## 2. Results

### 2.1. Chr23-miR-200s Knockout Leads to the Impaired Reproduction and Increased Growth in Female Zebrafish

**Figure 1:**
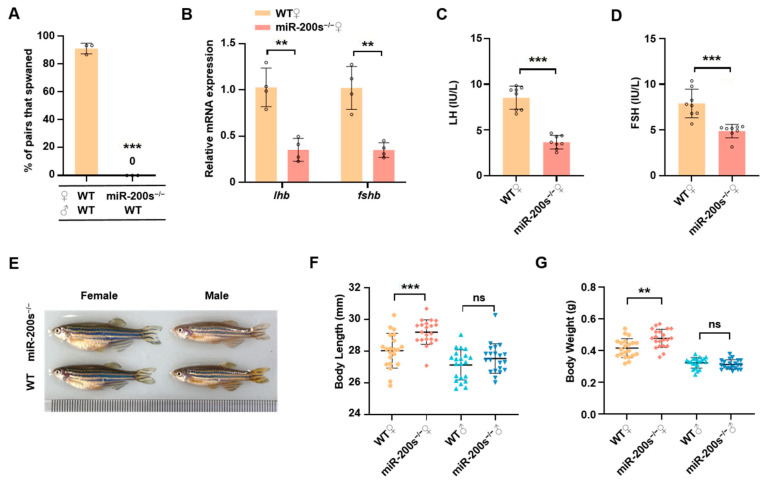
Impaired reproduction and promoted growth in female chr23-miR-200s-KO zebrafish. (**A**) Successful spawning rate of WT and chr23-miR-200s-KO (miR-200s^−/−^) females mating naturally with WT males. There were three independent repetitions (*n* = 15 for each). (**B**) mRNA expression *lhb* and *fshb* in the pituitary of female zebrafish. (**C**,**D**) LH and FSH levels in plasma of adult female zebrafish (*n* = 8). (**E**) Representative morphological images of WT and miR-200s^−/−^ zebrafish. (**F**,**G**) Statistics of body length and body weight of 3-month-old miR-200s^−/−^ and WT zebrafish (*n* = 22). Data are represented as mean ± SD (*p* < 0.01 **; *p* < 0.001 ***; ns, no significant).

Previous studies indicate that the male chr23-miR-200s-KO zebrafish had a higher fertilization rate than male WT zebrafish when they were crossed with female WT zebrafish by natural spawning [46]. In contrast, mating failure of the female chr23-miR-200s-KO zebrafish was observed in natural spawning (Figure 1A). To further understand the cause of female reproductive abnormality, we examined the expression of gonadotropins in the female pituitary gland. We found that the mRNA expression levels of *lhb* and *fshb* in pituitary, as well as the plasma levels of LH and FSH, were significantly reduced in female chr23-miR-200s-KO zebrafish compared with female WT zebrafish (Figure 1B–D). Additionally, after comparing the growth traits between WT and chr23-miR-200s-KO zebrafish, we found that the body length and body weight were significantly increased in the female chr23-miR-200s-KO zebrafish, but not significantly changed in male chr23-miR-200s-KO zebrafish (Figure 1E–G). These results reveal that zebrafish chr23-miR-200s regulate reproduction and growth in a sexually dimorphic manner, especially maintaining normal spawning of female zebrafish and controlling their body growth.

### 2.2. Chr23-miR-200s Regulate Growth Trait of Female Zebrafish by Directly Targeting stat5b

**Figure 2:**
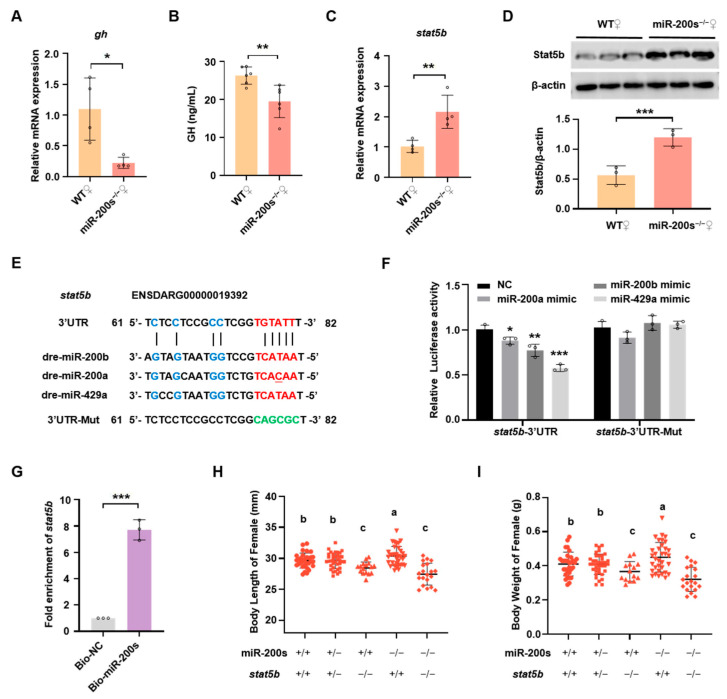
Chr23-miR-200s regulate growth of female zebrafish through targeting *stat5b* 3′UTR. (**A**) QRT-PCR analysis of *gh* expression levels in pituitary of WT and chr23-miR-200s-KO (miR-200s^−/−^) female zebrafish. (**B**) ELISA analysis of plasma GH in miR-200s^−/−^ and WT female zebrafish. (**C**) *Stat5b* mRNA expression levels in liver of female fish. (**D**) Western blot analysis of Stat5b expression in liver. Representative images (top) and the quantitation (bottom; *n* = 3 per assay). (**E**) MiR-200b/200a/429a seed region and predicted target sites within 3′UTR of *stat5b*, and the mutated 3′UTR sequence of *stat5b* (3′UTR-Mut). The seed sequence pairing regions are marked in red, while other pairing base sequences are marked in blue, and the green bases indicate the mutated pairing region in 3′UTR of *stat5b*. (**F**) Validation of potential target of miR-200s by a dual-luciferase reporter assay in HEK-293T cells. NC stands for the negative control. Data are represented as mean ± SD (*p* < 0.05 *, *p* < 0.01 **; *p* < 0.001 ***). (**G**) Fold enrichment of *stat5b* by using qRT-PCR in the sample pulled down by biotinylated miR-200s (miR-200b/-200a/-429a) and negative control (NC). (**H**,**I**) Statistics of body length (**H**) and body weight (**I**) of female self-bred offspring of chr23-miR-200s-KO and *stat5b* double heterozygotes at 3 mpf. The groups with different letters indicate that they are statistically significant (*p* < 0.05).

To elucidate the molecular mechanism of chr23-miR-200s regulating body growth in female zebrafish, we check the expression of some key genes related to body growth, including *gh* and *stat5b*. *Stat5b* has been revealed to regulate growth sex dimorphism in zebrafish [47]. Unexpectedly, the *gh* mRNA expression in pituitary was significantly lower in female chr23-miR-200s-KO zebrafish compared to female WT zebrafish (Figure 2A), which was further confirmed by a reduced level of GH in the plasma of chr23-miR-200s-KO zebrafish (Figure 2B). On the contrary, we found up-regulated mRNA and protein expression of *stat5b* in the liver of chr23-miR-200s-KO females (Figure 2C,D). To verify whether *stat5b* was a putative target gene of chr23-miR-200s (miR-200b, -200a and -429a), we used the bioinformatics algorithm to predict the binding site of chr23-miR-200s in the 3′UTR of *stat5b* (Figure 2E). Analysis of luciferase reporter assays shows that chr23-miR-200s significantly repressed the luciferase activity of *stat5b*-3′UTR plasmid, whereas mutation of the predicted chr23-miR-200s binding sites abolished the repressions (Figure 2F). Furthermore, we applied a biotin–avidin pull-down system to examine whether chr23-miR-200s could pull down the 3′UTR of *stat5b* in the liver of female zebrafish. As shown in Figure 2G, the 3′UTR of *stat5b* was pulled down by chr23-miR-200s as analyzed by qRT-PCR. Compared with the negative control, there was a 7.8-fold enrichment of *stat5b* 3′UTR in the chr23-miR-200s pull-down samples.

To further investigate whether *stat5b* functions as an important downstream regulator of chr23-miR-200s, double heterozygous fish (miR-200s^+/−^; *stat5b*^+/−^) were generated by crossing the chr23-miR-200s^+/−^ and *stat5b*^+/−^ zebrafish, which were intercrossed to produce several lines including the homozygous double-mutant line (miR-200s^−/−^; *stat5b*^−/−^). After genotyping, the body length and body weight of female zebrafish were examined in these lines (Figure 2H,I). There was no significant difference in growth between the doubly heterozygous fish (miR-200s^+/−^; *stat5b*^+/−^) and WT (miR-200s^+/+^; *stat5b*^+/+^) fish. The loss of *stat5b* in chr23-miR-200s homozygous mutant (miR-200s^−/−^; *stat5b*^−/−^) completely abolished the elevated growth performance induced by chr23-miR-200s deficiency (miR-200s^−/−^; *stat5b*^+/+^), and no significant difference in the growth performance was observed between the double-mutant (miR-200s^−/−^; *stat5b*^−/−^) and single-mutant (miR-200s^+/+^; *stat5b*^−/−^) zebrafish. Taken together, these data demonstrate that chr23-miR-200s regulate body growth of female zebrafish by directly targeting *stat5b* in a GH-independent manner.

### 2.3. Dmrt1 Knockout Results in Impaired Reproduction and Promoted Growth in Male Zebrafish

**Figure 3:**
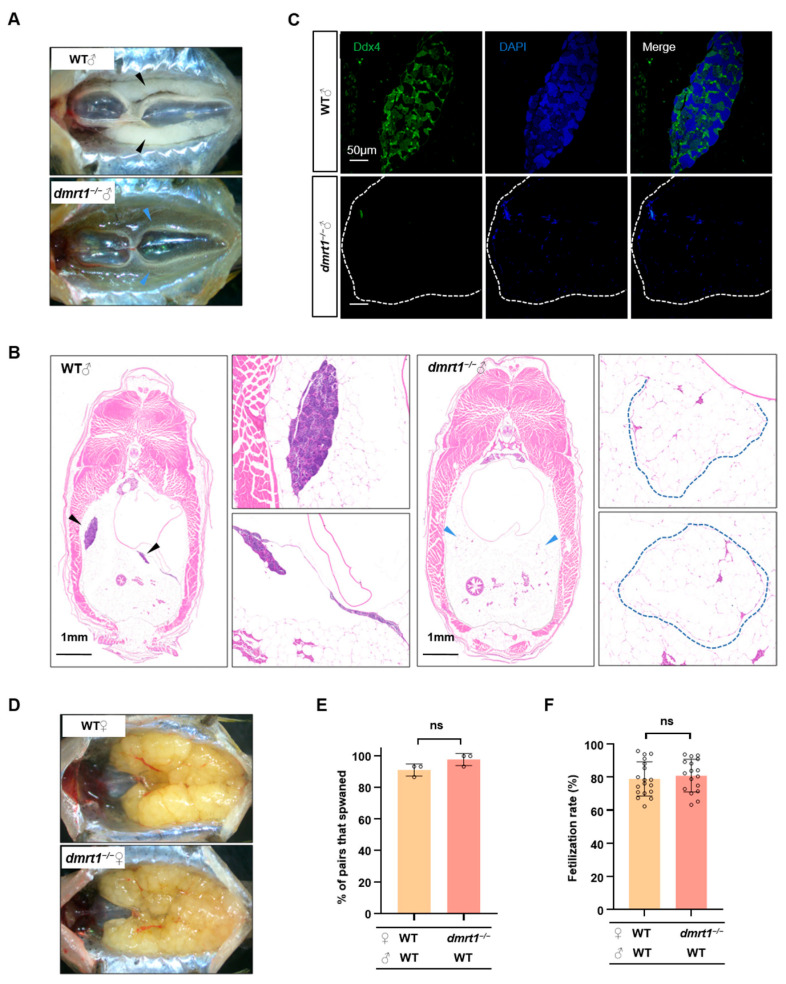
*Dmrt1* regulates gonad development in males but not in females. (**A**) Anatomical observation of testes in WT and *dmrt1*^−/−^ at 5 mpf. The black and blue arrowheads indicate the testes in WT and *dmrt1*^−/−^ zebrafish, respectively. (**B**) Histological examination of testes in 5-month-old WT and *dmrt1*^−/−^ zebrafish. Transverse section of the abdomen by HE staining. The black and blue arrowheads indicate the testes in WT and the severely regressed testicular tissue in *dmrt1*^−/−^ zebrafish, while the blue dotted lines show the amplified images of testes in *dmrt1*^−/−^ zebrafish. Scale bars, 1 mm. (**C**) Immunofluorescence staining of WT and *dmrt1*^−/−^ testis transverse sections with Ddx4 antibody (green). The nuclei were stained by DAPI (blue). The white dotted lines show the testes in *dmrt1*^−/−^ zebrafish. Scale bars, 50 μm. (**D**) Anatomical observation of ovary in 5-month-old WT and *dmrt1*^−/−^ zebrafish. (**E**) Successful spawning rate of WT and *dmrt1*^−/−^ females mating naturally with WT males at 5 mpf. There were three independent repetitions (*n* = 15). (**F**) Fertilization rate of WT and *dmrt1*^−/−^ females by crossing with WT males at 5 mpf (*n* = 15). Data are represented as mean ± SD (ns, no significant).

**Figure 4:**
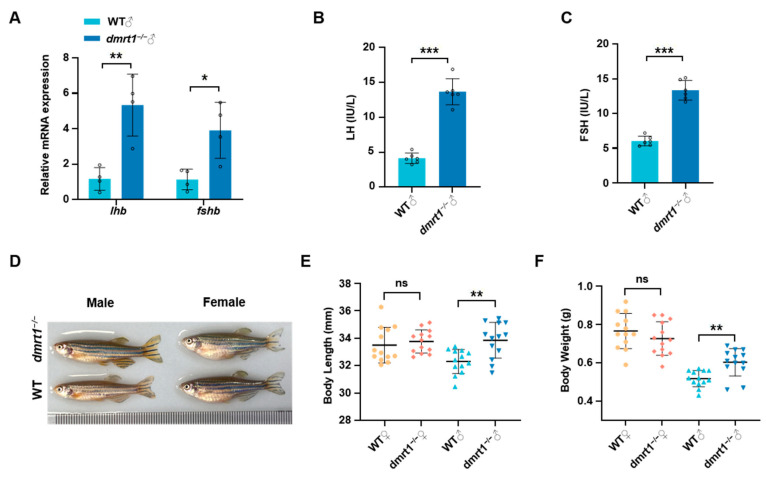
*Dmrt1* knockout leads to impaired reproduction and promoted growth in male zebrafish. (**A**) mRNA expression of *lhb* and *fshb* in the pituitary of WT and *dmrt1*^−/−^ male zebrafish. (**B**,**C**) LH and FSH levels in plasma of adult male zebrafish (*n* = 6). (**D**) Morphological comparison of WT and *dmrt1*^−/−^ zebrafish at 5 mpf. (**E**,**F**) Statistics of body length and body weight of WT and *dmrt1*^−/−^ zebrafish at 5 mpf (*n* = 13). Data are represented as mean ± SD (*p* < 0.05 *, *p* < 0.01 **; *p* < 0.001 ***; ns, no significant).

Our previous studies have shown that *dmrt1* is a key factor in the maintenance and self-renewal of male germ cells in zebrafish [46]. At 5 months-post-fertilization (mpf), all *dmrt1*^−/−^ male zebrafish displayed severely degenerated testes (Figure 3A). The degenerated testicular structure of adult male *dmrt1*^−/−^ can be clearly observed by HE staining of transverse section of abdomen (Figure 3B), and with very few germline cells labeled by Ddx4 signals (Figure 3C). Different from *dmrt1*^−/−^ male zebrafish, *dmrt1*^−/−^ female zebrafish had normal ovarian development and reproductive ability compared with WT females (Figure 3D–F). We found that the mRNA expression levels of *lhb* and *fshb* were significantly up-regulated in pituitary of *dmrt1^−^*^/*−*^ males (Figure 4A), which were further supported by the plasma levels of LH and FSH measured by ELISA (Figure 4B,C). In addition, after comparing the growth traits between WT and *dmrt1*^−/−^ zebrafish, we observed that the body length and body weight were significantly increased in the male *dmrt1*^−/−^ zebrafish, but not significantly affected in female *dmrt1*^−/−^ zebrafish (Figure 4D–F). These data suggest that zebrafish *dmrt1* regulates reproduction and growth in a sexually dimorphic manner, especially by controlling testis development and body growth in male zebrafish.

### 2.4. Dmrt1 Controls Somatic Growth by Transcriptionally Mediating stat5b Expression in Male Zebrafish

**Figure 5:**
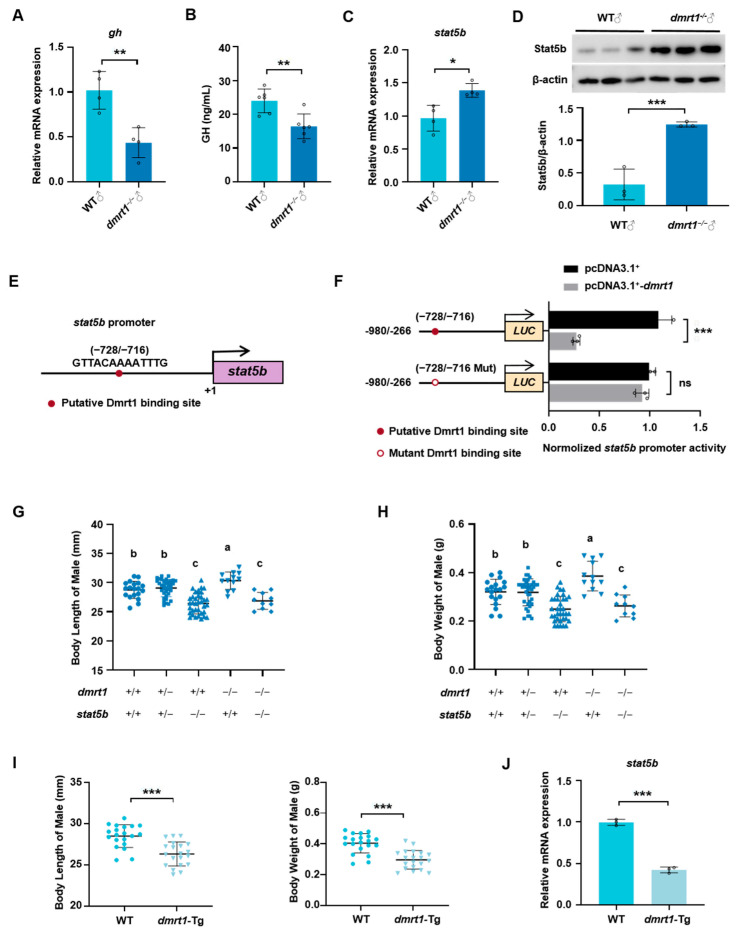
Dmrt1 regulates somatic growth in male zebrafish by mediating *stat5b* expression. (**A**,**B**) *Gh* mRNA levels in pituitary (**A**) and plasma GH levels (**B**) of WT and *dmrt1*^−/−^ male zebrafish at 5 mpf. (**C**,**D**) Relative expression of *stat5b* mRNA (**C**) and Stat5b protein (**D**) in liver. Representative images of Western blot (top) and the quantitation (bottom; *n* = 3 per assay). (**E**) Binding site of transcription factor Dmrt1 in the *stat5b* promoter region predicted by JASPAR. (**F**) Schematic representation of the normal and mutational *stat5b* promoter luciferase constructs and their activities in dual-luciferase assay. *Dmrt1* overexpression inhibited the luciferase activity of *stat5b* promoter in HEK-293T cells. The empty vector pcDNA3.1^+^ and pGL3-basic were transfected as the control. (**G**,**H**) The body length and body weight of male self-bred offspring of *dmrt1* and *stat5b* double heterozygotes at 3 mpf. The groups with different letters indicate that they are statistically significant (*p* < 0.05). (**I**) The body length and body weight of male *dmrt1* transgenic line at 3 mpf (*n* = 20). (**J**) Relative expression of *stat5b* mRNA in liver of *dmrt1* transgenic male fish. Data are represented as mean ± SD (*p* < 0.05 *, *p* < 0.01 **; *p* < 0.001 ***; ns, no significant).

In order to reveal the regulatory mechanism of *dmrt1* on body growth, we detected the mRNA transcriptional levels and plasma levels of GH, and found that they were significantly reduced in male *dmrt1*^−/−^ zebrafish compared to the WT (Figure 5A,B). In contrast, both mRNA and protein expression of *stat5b* were simultaneously up-regulated in male zebrafish when there was a loss of *dmrt1* function (Figure 5C,D). In addition, a transcription binding site of Dmrt1 was found in the proximal promoter region of *stat5b* (Figure 5E). The results of the dual-luciferase assay proved that Dmrt1 could directly transcriptionally regulate the promoter activity of *stat5b* (Figure 5F). Dmrt1 inhibited the luciferase activity of *stat5b* promoter in HEK-293T cells, and the inhibitory effect was abolished when the Dmrt1 binding site was mutated. Doubly heterozygous fish (*dmrt1*^+/−^; *stat5b*^+/−^) were generated by crossing the female *dmrt1^+/−^* and male *stat5b^+/−^* zebrafish, which were intercrossed to produce several lines, including the homozygous double-mutant line (*dmrt1*^−/−^; *stat5b^−/−^*). The body length and body weight of male zebrafish were examined in these different genotypes of lines (Figure 5G,H). There was no significant difference in growth between the double heterozygous fish (*dmrt1^+/−^*; *stat5b^+/−^*) and WT (*dmrt1^+/+^*; *stat5b^+/+^*) fish. The loss of *stat5b* in *dmrt1* homozygous mutant (*dmrt1*^−/−^; *stat5b^−/−^*) completely abolished the elevated growth performance induced by *dmrt1* deficiency (*dmrt1*^−/−^; *stat5b^+/+^*), and no significant difference in the growth performance was observed between the double-mutant (*dmrt1*^−/−^; *stat5b^−/−^*) and single-mutant (*dmrt1*^+/+^; *stat5b^−/−^*) zebrafish.

Furthermore, we generated a *dmrt1* ectopically overexpressed zebrafish line Tg (β-actin: *dmrt1-HA*). Body length and body weight were measured at 3 mpf, and the results show that *dmrt1*-overexpressed males had significantly lower body length and body weight than the WT males (Figure 5I), accompanied by a significant reduction in *stat5b* expression in the liver of transgenic fish (Figure 5J). Summarily, *stat5b* functions as an important downstream regulator of *dmrt1* on zebrafish body growth.

## 3. Discussion

Reproduction and growth, while superficially appearing as distinct biological processes, are in fact intricately interconnected. All biological processes necessitate substantial energy expenditure, and since the resources of animals (such as food intake) are finite, energy must be allocated among growth, reproduction, and maintenance of vital activities (such as metabolism and immunity) [48]. When energy is preferentially allocated to growth, reproductive activities may be suppressed; conversely, when energy is primarily devoted to reproduction, growth rates may decelerate. For instance, many animals undergo a rapid growth phase prior to sexual maturity, after which energy allocation shifts more towards reproductive activities [49]. The energy allocation theory is frequently invoked to elucidate the mechanisms underlying such trade-offs, particularly under conditions of caloric restriction, where these trade-offs become markedly pronounced. In this study, we utilized zebrafish as a model organism to investigate the relationship between reproduction and growth, as well as the underlying genetic regulatory mechanisms. Our findings demonstrate that chr23-miR-200s and Dmrt1 serve as key regulators of the trade-off between reproduction and growth in female and male zebrafish, respectively.

There exists a complex interplay between hormones and neuroendocrine factors that regulate reproduction and growth. For example, growth hormone (GH) not only participates in growth regulation but also plays a significant role in reproductive regulation. Research has demonstrated that GH deficiency can lead to hypogonadism, thereby impairing reproductive function [50]. GH transgenic fish display a delay of gonadal development and impaired reproduction in zebrafish and common carp [27,51]. Leptin, a signaling molecule indicative of energy status, acts on the hypothalamus to promote the secretion of GnRH, thereby regulating gonadal function [52,53]. In addition, after gonadectomy, the growth of tilapia was inhibited by decreased sex steroid levels [54]. These studies robustly illustrate that the regulatory mechanisms governing reproduction and growth are inextricably linked, achieving a dynamic equilibrium through energy allocation and physiological regulatory networks.

The regulatory mechanism of sexual dimorphism in feeding and metabolism has been preliminarily revealed. Sex hormone estradiol modulates leptin sensitivity to control feeding and sexual dimorphism in diet-induced obesity via hypothalamic Cited1 [55], while sex hormone receptors transcriptionally regulate sexually dimorphic gene expression [56]. MiR-200a/-200b has been shown to make a potential contribution to sexual size dimorphism in yellow catfish by targeting *leptin* [57]. The molecular mechanism of sexual dimorphism in glucose and lipid metabolism is highly complex in animals [58]. Reciprocal regulation of ERα and miR-22 specifically regulates muscle lipid metabolism in male mice [59]. Glutaredoxin 1 protects mice against obesity and atherosclerosis under nutrient stress in a sex-specific fashion [60]. One polymorphism locus in Dmrt1 is associated with gut microbiota composition and body weight in chickens [61]. Further studies need to be performed to investigate whether there is a functional interaction among chr23-miR-200s, Dmrt1 and the above factors to regulate sexual dimorphism in growth and metabolism.

Somatic growth in vertebrates is usually regulated by the GH/IGF signaling pathway in the hypothalamus–pituitary–liver (HPL) axis and its interaction with feeding regulation and metabolism [62]. MiR-200s have been shown to control zebrafish embryo size by targeting several GH/IGF axis genes, such as *GH*, *GHRa*, *GHRb*, and *IGF2a* [40]. However, in adult zebrafish, the RNA transcriptional levels and plasma levels of GH were significantly reduced in both chr23-miR-200s-KO females and *dmrt1*^−/−^ males with increased somatic growth, suggesting there is a GH-independent manner for somatic growth that has been reported previously [63,64]. Transgenic zebrafish with constitutively activated GHR (CA-GHR) show a higher growth rate, which does not rely on GH signaling activation [65]. STAT5b is the main downstream signaling of GH that plays an important role in regulating body growth in mammals [66]. Sexual dimorphism in growth has been revealed to be regulated by Stat5b in zebrafish, since *stat5b* deficiency in zebrafish results in severe growth retardation and diminishes the sexual growth dimorphism, while transgenic expression of *stat5b* promotes somatic growth [47,67]. *Stat5b* is a direct target of chr23-miR-200s and Dmrt1, while loss of *stat5b* function can completely abolish the elevated growth performance caused by chr23-miR-200s or *dmrt1* deficiency (Figure 2 and Figure 4), suggesting that chr23-miR-200s and Dmrt1 control sexual dimorphism in growth via their key downstream regulator *stat5b*.

## 4. Materials and Methods

### 4.1. Zebrafish Line and Maintenance

Zebrafish were maintained in a circulated water system at 28.5 °C on a 14 h light/10 h dark cycle. The wild-type line (AB) zebrafish was used in this study, while the zebrafish lines of homozygous *dmrt1* mutant (*dmrt1*^−/−^) [45], homozygous knockout of the miR-200 cluster on chromosome 23 (chr23-miR-200s-KO) [46], and homozygous *stat5b* mutant (*stat5b*^−/−^) [67] were as previously described. The double-knockout mutant heterozygotes were obtained by crossing the *stat5b*^+/−^ fish with *dmrt1*^+/−^ or chr23-miR-200s^+/−^ fish. Then, double-knockout heterozygotes were self-crossed to obtain the double-homozygous mutants (miR-200s^−/−^; *stat5b*^−/−^ and *dmrt1*^−/−^; *stat5b*^−/−^).

A transgenic zebrafish Tg (β-actin: *dmrt1-HA*) was generated via Tol2-mediated transgenesis. The zebrafish *dmrt1* ORF sequence and HA tag were cloned into pTol2 vector, and their expression was driven by zebrafish β-actin promoter [68]. The linearized recombinant pTol2 plasmid and transposase mRNA were co-injected into one-cell stage zebrafish embryo to obtain F0 generation. Protein of F0 embryos was extracted and HA antibody was used for Western blot to verify the expression of Dmrt1-HA fusion protein. Subsequently, DNA from tail was extracted for PCR screening of transgenic individuals. F1 generation was obtained by mating F0 with WT, then the screened F1 crossed again with WT to produce F2. By intercrossing F2 heterozygous individuals, a stable F3 generation of homozygous *dmrt1* transgenic zebrafish was established. Finally, the F3 generation was used for future studies.

All experimental fish for body length and body weight measurement were from heterozygous self-breed siblings, raised in the same tank, and genotype identification was performed before measurement.

### 4.2. Quantitative Real-Time PCR

Total RNA was extracted from pituitary or liver tissues of adult zebrafish using TRIzol reagent (Invitrogen, Carlsbad, CA, USA) and reverse-transcribed into cDNA using a PrimeScript RT reagent kit (TaKaRa, Beijing, China) according to the manufacturer’s protocol. Quantitative real-time PCR was performed using Hieff qPCR SYBR Green Master Mix Kit (Yeasen, Shanghai, China) and run on the CFX96 Touch™ Real-Time PCR Detection System (Bio-Rad, Hercules, CA, USA) as described previously [40]. The relative mRNA expression was normalized to the reference gene β-actin. The qRT-PCR primers are listed in Table S1. The data were analyzed using the 2^−ΔΔCt^ method.

### 4.3. ELISA Analysis of Plasma Hormone

Blood collection from zebrafish was conducted according to the method previously described [69]. Zebrafish were anesthetized with MS-222, then 10 µL of blood was extracted from the amputated tail for each fish. Blood samples were collected into 1.5 mL anticoagulant tubes and maintained at 4 °C. Blood from two fish was pooled to form one sample, with at least three biological replicates per group. The samples were then centrifuged at 5000× *g* for 20 min at 4 °C to collect the plasma supernatant. Plasma was transferred to sterile centrifuge tubes and stored at −20 °C until further analysis. The fish LH, FSH, and GH ELISA Kits (JSBOSSEN, Nangjing, China) were used for measurement following the manufacturer’s instructions. The standard with gradient concentrations and the samples to be tested were mixed with horseradish peroxidase (HRP)-conjugated antibody, incubated at 37 °C for 60 min, and washed with washing solution 5 times. After being added with the substrate and incubated at 37 °C in dark light for 15 min, the absorbance of the samples was measured at 450 nm after termination.

### 4.4. Western Blot

The liver tissues of the control and mutant zebrafish were homogenized and lysed in lysis buffer. The lysates were centrifugated at 12,000 rpm at 4 °C for 15 min, then protein concentration was measured by BCA Protein Assay Kit (CWBIO, Beijing, China). Equal amount of protein samples were separated in 10% SDS-PAGE gels and transferred onto 0.45 μm PVDF membranes (Bio-Rad, Hercules, CA, USA). After blocking, the membranes were incubated overnight at 4 °C with primary antibodies, rabbit polyclonal anti-Stat5b antibody (1:1000, #A21613; ABclonal, Wuhan, China), or rabbit monoclonal anti-β-actin antibody (1:100,000, #AC026; ABclonal, Wuhan, China). The blots were detected with horseradish peroxidase (HRP)-conjugated goat anti-rabbit IgG secondary antibody and visualized using an enhanced chemiluminescence (ECL) detection reagents kit (Bio-Rad, Hercules, CA, USA). The blots were scanned by ImageQuant LAS 4000 software (version 1.2)and quantified by NIH software Image J (version 1.8.0).

### 4.5. Histological Analysis and Immunofluorescence Staining

Wild-type and *dmrt1*^−/−^ male zebrafish at 5 mpf were euthanized with MS-222. The head and tail were dismembered and the abdominal cavity was fixed overnight with 4% paraformaldehyde (PFA). After decalcification and ethanol gradient dehydration, the tissues were embedded in paraffin wax and sectioned into 5 μm slides along cross section. Paraffin slides used for histological analysis were deparaffinized and rehydrated in PBS, and stained with hematoxylin and eosin (HE). Images were acquired using a Pannoramic SCAN system (3DHISTECH, Budapest, Hungary).

For immunofluorescence staining, the sections after deparaffinization were rehydrated in PBS, repaired in bath of 0.01 M citric acid antigen retrieval buffer (pH = 6) at 97 °C for 10 min. Then, slides were blocked with 5% BSA in PBS for 30 min at room temperature, and incubated overnight at 4 °C with rabbit polyclonal anti-Ddx4 (Vasa) primary antibody (1:100, #A15624; ABclonal, Wuhan, China). After washing 3 times with PBS, slides were incubated with fluorescein isothiocyanate (FITC) conjuganted goat anti-rabbit IgG secondary antibody at room temperature for 1 h. Finally, the nucleus was stained with DAPI for 10 min and washed 3 times with PBS. Images were acquired using TCS SP8 Leica confocal microscope (Leica, Nussloch, Germany).

### 4.6. Biotinylated miRNA Pulldown

The biotinylated RNA probes Bio-miR-200a/-200b/-429a (Bio-miR-200s) and Bio-NC were synthesized by GenePharma (Shanghai, China) and dissolved in DEPC water to 20 μM. Dynabeads M-280 Streptavidin (Invitrogen, Carlsbad, CA, USA) were preprocessed according to the manufacturer’s protocol, which were pre-blocked with RNase-free bovine serum albumin. Then, biotin-labeled RNA probes were incubated with beads at 4 °C overnight on a rotator to generate probe-coated beads. Adult zebrafish were euthanized with MS-222 and 50 mg of liver tissue was collected for each experiment. The tissue was homogenized in cold RNase-free cell lysis solution and centrifuged for 5000 rpm at 4 °C to collect the cell lysis supernatant. The processed cell lysate was divided into two parts and, respectively, incubated with Bio-miR-200s or Bio-NC probe-coated magnetic beads by rotating at 4 °C for 6 h. Subsequently, breads were washed 5 times by cold RNase-free lysis buffer. Finally, the RNA complexes bound to beads were purified with TRIzol for qRT-PCR analysis.

### 4.7. Luciferase Reporter Assay

The 3′ untranslated region (3′UTR) of *stat5b* contains a putative miR-200s binding site predicted by bioinformatics algorithms. The 3′UTR fragment of *stat5b* (*stat5b*-3′UTR) containing a putative miR-200s binding site and the *stat5b*-3′UTR with binding site mutation (*stat5b*-3′UTR-Mut) were synthesized (Tsingke, Beijing, China) and inserted into the pmir-GLO vector (Ambion, Austin, TX, USA). MiR-200a/-200b/-429a mimics and their negative control were synthesized by GenePharma (Shanghai, China). HEK-293T cells were cultured in 24-well plates and co-transfected with 25 ng constructed pmir-GLO plasmid (*stat5b*-3′UTR or *stat5b*-3′UTR-Mut) and 50 nM miR-200a, miR-200b, miR-429a mimic or negative control (NC) using FuGENE HD Transfection Reagent (Promega, Madison, WI, USA) [40]. Twenty-four hours after transfection, the cells were collected and measured using luciferase activities using the Dual Luciferase Reporter Assay System (Promega, Madison, WI, USA) according to the manufacturer’s instructions. Relative *Firefly* luciferase activity was normalized with *Renilla* luciferase activity. Each experiment was conducted in triplicate.

The JASPAR database (https://jaspar.genereg.net/, accessed on 13 December 2022) was employed for promoter prediction, and a putative Dmrt1 binding site was found in *stat5b* promoter region (−728/−716). The promoter sequence (−1246/−267, 980 bp) of *stat5b* were cloned and inserted into pGL3-basic plasmid. The mutated *stat5b* promoter sequence was generated by replacing the Dmrt1 binding site “TACAAAATT” with “TTGCGAATT”, using Mut ExpressII Fast Mutagenesis Kit (Vazyme, Nanjing, China) according to the manufacturer’s protocol. The ORF of *dmrt1* was amplified and inserted into pcDNA3.1^+^ vector (Invitrogen, Carlsbad, CA, USA). HEK-293T cells were cultured according to the previous report, cells were seeded in 24-well plates and co-transfected with recombinant plasmid pGL3-*stat5b*, pcDNA3.1^+^-*dmrt1* and pRL-TK at a ratio of 10:10:1 using FuGENE HD Transfection Reagent. The empty vector pcDNA3.1^+^ and pGL3-basic were transfected as the control. Twenty-four hours post-transfection, the cells were collected to measure luciferase activity. Relative *Firefly* luciferase activity was normalized with *Renilla* luciferase activity. Primer information for plasmid construction is listed in Table S1.

### 4.8. Statistical Analysis

All of the results are shown as the mean ± standard deviation. Each result represents the mean of at least three independent experiments. To compare the differences between two groups and more groups, two-tailed unpaired Student’s *t*-tests and one-way ANOVA with post hoc contrasts by Duncan’s test were calculated with Prism software 8. A probability (*p*) of *p* < 0.05 *, *p* < 0.01 **, and *p* < 0.001 *** was considered statistically significant.

## 5. Conclusions

In this study, we found that chr23-miR-200s specifically regulates the trade-off between reproduction and growth in female zebrafish. In contrast, Dmrt1 specifically regulates the trade-off between reproduction and growth in male zebrafish. We provided a research model for further investigation on regulatory mechanism of sexually dimorphic trade-off between reproduction and growth in vertebrates.

## Data Availability

Data is contained within the article and Supplementary Materials.

## Supplementary Materials

The following supporting information can be downloaded at: https://www.mdpi.com/article/10.3390/ijms26041785/s1.
