# Supplementary material for: Chr23-miR-200s and Dmrt1 Control Sexually Dimorphic Trade-Off Between Reproduction and Growth in Zebrafish
Source: Int J Mol Sci. 2025 Feb 19;26(4):1785. doi: 10.3390/ijms26041785 (PMC11855846; doi:10.3390/ijms26041785)
Supplement: Supplementary file 1 [file ijms-26-01785-s001.zip › ijms-3402898-supplementary.pdf]

Table S1. qRT-PCR and plasmid construction.

Gene name	Primer sequence (5′-3′)	Purpose
lhb-F	ATGTTATTGGCTGGAAATGG	qRT-PCR
lhb-R	CTAGTATGCGGGGAAATCC	
fshb-F	TGGACAAGATACGCTGAAGCAA	qRT-PCR
fshb-R	GCAAGTGGCTTCTGATGTGATG	
stat5b-F	CCTGAAGCCTCACTGGAATG	qRT-PCR
stat5b-R	GAGTCGCTGAATCGCAACA	
β-actin-F	CGAGCAGGAGATGGGAACC	qRT-PCR
β-actin-R	CAACGGAAACGCTCATTGC	
stat5b promoter-F (Kpn I)	ggggtaccAAGGTCAAGGTGCTCAAGGA	Plasmid construction
stat5b promoter-R (Xma I)	cccccgggGGTTTGGAAGAAGTGAAGGGATA	
dmrt1 ORF-F (BamH I)	cgggatccATGAGTGAAGAAGAGCAGACTA	Plasmid construction
dmrt1 ORF-R (EcoR I)	cggaattcTCATTTGGTGGCCCCCTCTATGA	
dmrt1 ORF-HA-F (Xma I)	ccccgggATGAGTGAAGAAGAGCAGACTA	Construction of Tg (β - actin:*dmrt1-HA*)
dmrt1 ORF-HA-R (Kpn I)	ggggtaccTCAAGCGTAATCTGGAACATCGTATGGGTATTTGGTGGCCCCCTCTATG	
dmrt1 Tg-F	ATGAGTGAAGAAGAGCAGACTAACGGG	*dmrt1*-Tg screening
dmrt1 Tg-R	TCAAGCGTAATCTGGAACATCGTATG	
